# Evaluation of the Safety of Percutaneous Dilatational Tracheostomies in Patients with Antiplatelet Therapy—A Comparison of Two Single-Step Percutaneous Dilatational Techniques

**DOI:** 10.3390/jcm14145036

**Published:** 2025-07-16

**Authors:** Lukas Ley, Mustafa Kerem Cinar, Anita Windhorst, Jens Allendoerfer, Hossein Ardeschir Ghofrani, Dirk Bandorski

**Affiliations:** 1 Campus Kerckhoff, Justus-Liebig-University Giessen, 61231 Bad Nauheim, Germany; lukas.m.ley@med.uni-giessen.de; 2 Asklepios Neurologische Klinik Bad Salzhausen, 63667 Nidda, Germany; m.cinar@asklepios.com (M.K.C.); j.allendoerfer@asklepios.com (J.A.); 3 Institute for Medical Informatics, Justus-Liebig-University, 35392 Giessen, Germany; anita.windhorst@informatik.med.uni-giessen.de; 4 Department of Internal Medicine, Justus-Liebig-University Giessen, Universities of Giessen and Marburg Lung Center (UGMLC), 35392 Giessen, Germany; h.ghofrani@kerckhoff-klinik.de; 5 Department of Pneumology, Kerckhoff Heart and Thorax Center, 61231 Bad Nauheim, Germany; 6 Department of Medicine, Imperial College London, London SW7 2AZ, UK; 7 Faculty of Medicine, Semmelweis University Campus Hamburg, Lohmühlenstraße 5, 20099 Hamburg, Germany

**Keywords:** tracheostomy, percutaneous dilatational tracheostomy, PDT, antiplatelet therapy, APT

## Abstract

**Introduction:** Antiplatelet therapy (APT) increases bleeding risk and is frequently used in patients who undergo percutaneous dilatational tracheostomy (PDT). However, there are different techniques for single-step PDTs, which can be differently invasive. The aim of the present study was to investigate complications in patients undergoing PDT while being on APT, especially with regard to bleeding and the influence of different PDT techniques. **Material and Methods:** Between July 2016 and June 2021, 273 intensive care unit (ICU) patients underwent in-house PDT with two different techniques (direct or indirect) and were retrospectively enrolled. **Results:** A total of 273 patients (mean age: 68 years, 37% female) were included in the study. A total of 51% of patients were on APT on the day of PDT procedure (SAPT: 34%, DAPT: 17%). Direct and indirect PDTs were performed in 33% and 67% of patients. Periprocedural airway or skin bleedings and postprocedural bleedings occurred in 53%, 11%, and 1%. A need for bronchoscopic re-intervention was observed in 2% of APT patients. No death was procedure related. Periprocedural airway bleedings occurred more frequent in “APT patients” (60% vs. 46%, *p* = 0.03). Periprocedural airway and skin bleedings were more frequent in indirect PDTs (52% and 14%) than direct PDTs (32% and 0%, *p* = 0.04 and *p* = 0.02) in “no APT patients”. In “APT patients” this difference was only seen in periprocedural airway bleeding (69% vs. 45%, *p* = 0.01). Moreover, periprocedural airway bleedings were more frequent in “APT patients” when performing an indirect PDT rather than a direct PDT (69% vs. 52%, *p* = 0.02). **Conclusions:** PDTs appear to be safe in patients receiving APT. Indirect PDTs appear to generally increase the risk of clinically irrelevant, minor periprocedural airway and possibly skin bleedings, especially in APT patients.

## 1. Introduction

Antiplatelet therapy (APT) is frequently used, e.g., in patients with acute coronary syndrome, ischemic stroke, and transient ischemic attack [1,2,3]. It is not uncommon that these patients are critically ill, are treated in intensive care units (ICUs), and require long-term ventilation. Because percutaneous dilatational tracheostomies (PDTs) are advantageous for long-term ventilation and subsequent respiratory weaning, they are an established and regularly performed procedure in ICUs [4,5,6,7]. However, there are various different PDT techniques, e.g., direct and indirect single-step PDT [7,8]. Moreover, because APT increases bleeding risk [9], there is a theoretically increased risk of bleeding and possibly other complications when performing a PDT in those patients. Some authors even consider (significant) coagulopathies as (relative) contraindications for PDT [10,11]. Although there are a few studies that have investigated this potential risk [12,13,14,15,16,17,18,19,20,21,22,23], there have not been any studies comparing different PDT techniques in this context yet. From our own experience, the indirect technique, at which the performing physician first dissects the pre-tracheal tissue before puncturing and dilatating the trachea, is associated with larger skin incisions and wound areas, appears more invasive overall, and the bleedings are more difficult to control. Therefore, the aim of the present study was to investigate the frequency, severity, and management of bleedings and other complications in patients who underwent PDT while receiving APT, especially with regard to the influence of different PDT techniques.

## 2. Material and Methods

### 2.1. Study Design

The present study was conducted as a unicentre, retrospective study in the ICU of a German neurological specialist hospital. Between July 2016 and June 2021, consecutive patients were retrospectively analyzed regarding the inclusion criteria (in-house PDT, ≥18 years of age, and available written informed consent to participate in the study) and were included if they met all these criteria. Corresponding clinical data was then extracted from the hospital’s data system, anonymized, and analyzed. The study was conducted according to the guidelines of the Declaration of Helsinki and approved by the Institutional Review Board of the Justus Liebig University Giessen (#177/21, dated 1 October 2021).

### 2.2. Definition of Parameters

Various clinical parameters were included in the present study. While many of these are standard parameters, some others need to be explained. “APT patients” were all patients receiving APT, either single APT (SAPT) or dual APT (DAPT), during the PDT procedure. “No APT patients” comprised all patients not receiving any APT during the PDT procedure. Death in ICU or hospital comprised various causes of death (all-cause death). Periprocedural airway bleeding was defined as a bleeding in the patient’s airway of any severity during or directly after the PDT procedure. Periprocedural skin bleeding was defined as a superficial bleeding of any severity near the tracheostomy site during or directly after the PDT procedure. Postprocedural bleeding was defined as any bleeding of any severity probably linked to the PDT procedure (airway or skin bleeding). Tracheal cartilage fracture was detected by tracheoscopy. Re-intervention was defined as any procedure (e.g., bronchoscopy) that had to be carried out because of any complications linked to the PDT procedure. Fever was defined as a rectal temperature ≥ 38 °C.

### 2.3. Tracheostomy Procedure

The PDT techniques used have been described before [7]. All PDTs (Tracoe Experc Tracheostomy Kit; TRACOE medical GmbH, Nieder-Olm, Germany) were performed bedside as direct or indirect single-step PDTs according to Ciaglia’s technique with simultaneous bronchoscopy [8,24,25]. Before and during the whole procedure, all patients were ventilated with FiO_2_ = 1.00. All patients were placed in a supine position, with a pillow placed under the shoulders and/or upper back to achieve sufficient neck extension. After sterilization, the puncture site was chosen based on anatomical landmarks. Following the administration of a local anesthesia (lidocaine), the endotracheal tube was deflated and retracted, the bronchoscope was inserted, and the puncture site was marked using diaphanoscopy. Direct PDTs were then performed as follows: after puncturing the trachea between the 1st and 2nd, or the 2nd and 3rd tracheal ring under aspiration and bronchoscopic control, a Seldinger guidewire was inserted, followed by a skin incision and targeted preparation along the guidewire. After insertion of a single-step conical dilator, the tracheal cannula was placed. On the contrary, indirect PDTs were performed as follows: after the skin incision and blunt dissection the trachea was punctured between the 1st and 2nd, or the 2nd and 3rd tracheal ring under aspiration and bronchoscopic control, and a Seldinger guidewire was inserted. After the insertion of a single-step conical dilator, the tracheal cannula was placed [8]. After confirming the correct cannula localization by bronchoscopy, the endotracheal tube was then completely removed, the tracheostomy cuff inflated, and the tracheostomy tube fixed and connected to the ventilator. All patients received fentanyl or sufentanil, propofol, and cisatracurium as a short-acting muscle relaxant. Discontinuation of therapeutic anticoagulation (TAC) and APT was at the discretion of the treating physician considering individual bleeding and thromboembolism risks. Both did not necessarily have to be stopped.

### 2.4. Statistical Analysis

Statistical analysis was performed using R (version 4.4.3) [26]. Descriptive statistics for categorial variables are the number of observations and percentages and associations with tah were tested using Fisher’s exact test or Fisher’s test, with simulation of *p*-values as indicated in the respective tables. Metric parameters are described using mean and standard deviations, median, and the interquartile interval. Differences between groups with and without APT are tested using Wilcoxon’s rank sum test. Tables are built using the R-package *Hmisc* [27]. Subgroup analysis was performed with technique of tracheotomy. Differences in different parameters over time were analyzed with a linear mixed model with repeated measures using the R package nlme [28]. Pairwise comparisons were calculated using emmeans for post hoc pairwise differences [29]. Model summary parameters are calculated and displayed using modelsummary [30].

## 3. Results

A total of 273 patients (mean age: 68 years, 37% female) were included in the study (Table 1). A total of 51% of patients were receiving APT during the PDT procedure (“APT patients”). A total of 34% of patients were on SAPT on the day of PDT procedure. Acetylsalicylic acid (ASS, 100 mg: 99%, 200 mg: 1%) was used in 100% of patients as SAPT medication. Moreover, 17% of patients were on DAPT on the day of PDT procedure. DAPT combinations were ASS 100 mg + clopidogrel 75 mg or 300 mg (61% and 1%), ASS 100 mg + ticagrelor 180 mg (30%), or ASS 100 mg + prasugrel 10 mg (8%). Additionally, 59% of patients were on prophylactic anticoagulation on the day of PDT procedure (65% of “no APT patients”, 54% of “APT patients”). Certoparin sodium was most commonly used for prophylactic anticoagulation (92%). Other drugs used for prophylactic anticoagulation were enoxaparin sodium (3%), dabigatran (3%), fondaparinux (1%), and unfractionated heparin (UFH, 1%). A total of 6% of patients (“no APT patients”: 4%, “APT patients”: 7%) were on TAC (enoxaparin sodium, UFH, argatroban, or apixaban) on the day of PDT procedure.

Indication for every tracheostomy was an expected prolonged ventilation. Direct PDTs were performed in 33% of patients (“no APT patients”: 29%, “APT patients”: 38%) and indirect PDTs were performed in 67% of patients (“no APT patients”: 71%, “APT patients”: 62%). Further procedural data can be found in Table 2.

Regarding the laboratory data, statistically significant differences were observed in hemoglobin, which was lower in “APT patients” (8.9 and 8.9 g/dL) than in “no APT patients” (9.5 and 9.3 g/dL, *p* = 0.004 and *p* = 0.02) preprocedure and three days postprocedure. Moreover, urea was statistically significantly higher in APT patients (76, 71 and 65 mg/dL) than in no APT patients (62, 57 and 54 mg/dL; all *p* < 0.001) preprocedure and one and three days postprocedure. Further laboratory data can be found in Table 3 but did not show any other significant anomalies or differences.

Observed complications of the PDT procedure (Table 4) comprised the following: periprocedural airway or skin bleeding (53% and 11%), postprocedural bleeding (1%), tracheal cartilage fracture (39%), and fever (23–40%). Statistically significant differences between “APT patients” and “no APT patients” were only seen in periprocedural airway bleedings, which occurred more frequent in “APT patients” (60% vs. 46%, *p* = 0.03). A need for (bronchoscopic) re-intervention was observed in 2% of APT patients. Moreover, a total of 34% patients (“no APT patients”: 30%, “APT patients”: 39%, *p* = 0.13) needed packed red blood cells (pRBCs) after PDT. The amount of pRBC units needed were not significantly different between groups (1: 3 vs. 4%, 2: 82 vs. 87%, 4: 12 vs. 7%, 6: 3 vs. 2%; *p* = 0.49–1.00). Furthermore, none of these bleedings were major and life-threatening and usually well controllable by local measures. No death was procedure related.

When comparing the direct and indirect PDT technique in patients with or without APT (Table 5) the following differences were statistically significant: periprocedural airway and skin bleedings were more frequent in indirect PDTs (52% and 14%) than direct PDTs (32% and 0%, *p* = 0.04 and *p* = 0.02) in “no APT patients”. In “APT patients” this difference was only seen in periprocedural airway bleeding (69% vs. 45%, *p* = 0.01). Moreover, periprocedural airway bleedings were more frequent in “APT patients” when performing an indirect PDT (69% vs. 52%, *p* = 0.02). There was no significant difference in the death in ICU or hospital rate, the days from PDT to death, postprocedural bleeding, tracheal cartilage fractures, mean pRBC units required after PDT, the need for bronchoscopic re-intervention, or fever on procedure day, and one or three days after PDT.

## 4. Discussion

The present study included 273 ICU patients (mean age: 68 years, 37% female) of which 51% were receiving APT (67% SAPT, 33% DAPT) during the PDT procedure. Additionally, 59% and 6% of patients were on prophylactic and/or therapeutic anticoagulation on the day of PDT procedure (Table 1). However, aPTT and quick values, as well as platelet count, were within reference ranges pre- and postprocedure with the INR only minimally elevated (Table 3). This indicates that prophylactic and therapeutic anticoagulation probably did not significantly contribute to a potential increase in the risk of bleeding.

Several complications were observed (Table 4). Periprocedural airway or skin bleeding and postprocedural bleeding were observed in 53%, 11%, and 1% of patients. These numbers may appear quite high, which was due to the applied definition of bleeding (any severity). The bleeding was not major or life-threatening in any patient and could be treated quickly and safely with local measures (pressure and gauze) in all patients. In line with this, there was no clinically relevant or significant hemoglobin drop after the PDT procedure. Moreover, a total of 34% of patients needed pRBCs after PDT. The number of patients requiring pRBCs and the amount of pRBC units needed were not significantly different between groups. The significantly lower hemoglobin and higher urea levels pre- and postprocedural in “APT patients”, as well as the insignificantly higher death in ICU rate, are most likely reflecting a higher morbidity in those patients. In line with this, pRBCs were not transfused because of procedure-related blood loss but because of preprocedural low hemoglobin levels. Only in 2% of APT patients was bronchoscopic diagnostic re-intervention necessary. No death was procedure related. A statistically significant difference in complications between “APT patients” and “no APT patients” was only seen in periprocedural airway bleedings, which occurred more frequently in “APT patients” (60% vs. 46%, *p* = 0.03). Lastly, the observed increased fever rates postprocedural (28% on procedure day vs. 40% one day postprocedural) are most likely due to aseptic, resorption fever and not procedure-related infections, as no clinical signs of infection at the PDT sites were observed. These findings indicate that there probably is no clinically relevant increased risk of bleeding when performing a PDT in patients with SAPT or DAPT. These observations are in line with the current study situation. However, there might be a clinically irrelevant increased risk of periprocedural airway bleeding in “APT patients” undergoing PDT.

### 4.1. Bleeding Risk of Percutaneous Dilatational Tracheostomy in Coagulopathic Patients

For the PDT procedure, the rate of clinically relevant bleedings is about 2–5%. Bleedings are very rarely major, and most are minor. In some studies, minor bleedings occur in 10–20% of patients [31,32,33,34,35].

APT and TAC are frequently used in ICU patients and lead to “iatrogenic coagulopathy” [1,20]. In addition, there are many other ICU patients with coagulopathies of various causes, such as thrombocytopenia, sepsis, trauma, liver disease, or the use of extracorporeal membrane oxygenation (ECMO) or ventricular assistant devices (VADs) [36,37]. Therefore, there is a theoretically increased risk of bleeding and possibly other complications when performing a PDT in those patients.

Studies in ECMO and VAD patients show an elevated bleeding risk (all bleeding: about 10–40%, major bleeding: 0–10%, and minor bleeding: 10–30%). However, most of the bleedings are minor and it was concluded that despite the increased bleeding risk PDT is safe and feasible in ECMO and VAD patients, especially if the coagulation status is optimized and anticoagulation held before the procedure [38,39,40,41,42,43,44,45,46,47,48,49]. In patients with “coagulopathies” of different causes, all bleedings, major bleedings, and minor bleedings occurred in about 1–20%, 0–5%, and 1–20%, respectively, also indicating a slightly elevated bleeding risk when performing a PDT [50,51,52,53,54,55,56,57,58]. However, there are also studies suggesting no statistically significant difference regarding the risk of bleeding in patients with “coagulopathies” [50,55,56,57]. Previous studies in patients with APT and/or AC use show a prevalence of about 2–15%, 0–5%, and 5–15% for all bleeding, major bleeding, and minor bleeding, respectively [12,13,14,15,16,17,18,19,20,21,22,23]. Therefore, the bleeding risk might not be significantly increased in patients with APT and/or AC, which is in line with the findings of the present study. When considering that bleeding definitions vary in most studies, and clinically irrelevant bleedings are sometimes not even considered a complication, our results fit in well with the current study situation (0% major, life-threatening, or clinically relevant bleedings; 50–60% clinically irrelevant bleedings which are well controllable with local measures).

However, it is important to note that the study situation on the bleeding risk in patients with any type of coagulopathy (ECMO, VAD, thrombocytopenia, severe liver disease, anticoagulation, or APT) undergoing PDT is difficult to evaluate conclusively, and all of the above figures can only be regarded as estimates as the corresponding studies are very heterogeneous. Many studies report on only a few patients and may therefore be underpowered and have difficulties detecting statistically significant differences between cohorts. In addition, the definitions of bleeding are usually not consistent. Furthermore, there are sometimes considerable differences in the preprocedural transfusion protocols (pRBCs, fresh frozen plasma, platelets, etc.) and the discontinuation protocol of heparin or other anticoagulants before the procedure. For this reason, the most relevant studies regarding the risk of bleeding in patients with anticoagulation and/or APT undergoing PDT will be discussed in the following paragraph.

Noy et al. [21] conducted a retrospective cohort study on the bleeding and mortality outcomes of PDT among critically ill patients receiving APT in 2024. They included 1662 patients (mean age: 61, 33% female). A total of 16.6% of patients received APT (13.1% aspirin, or 3.5% aspirin and clopidogrel). Rates of major and minor early (≤7 days) bleeding were 1.7% and 15.3% in the DAPT group (*n* = 59), 1.4% and 6.9% in the SAPT (*n* = 217), and 0.5% and 4.1% in the no APT group (*n* = 1386). Noy et al. found an increased bleeding risk for minor (OR: 4.22 and OR: 1.87) but not major early bleeding in DAPT and SAPT patients. Moreover, mortality was not increased in patients receiving SAPT or DAPT [21].

Luesebrink et al. [20] included 671 patients (mean age: 65, 34% female) in an international, multicenter, retrospective study on PDT in high-risk ICU patients in 2021. According to the prescribed drugs and dosages they formed the following four different groups: UFH prophylactic dosage (15%), UFH therapeutic dosage (20%), APT and UFH either prophylactic or therapeutic dosage except for triple therapy (43%), and triple therapy (DAPT with UFH in therapeutic dosage, 22%). UFH was held 4 h pre- and postprocedure. They observed procedure-related bleedings in 11% of patients, which were in almost all cases associated with skin bleeding from the entry site and could easily be treated with stitching. However, 1% of bleedings required CPR, which was carried out successfully. They found out that the occurrence of bleeding was not associated with any antithrombotic treatment regime. Luesebrink et al. concluded that “PDT was a safe and low-complication airway management option, even in a cohort of high risk for bleeding on cardiovascular ICUs” [20].

Lastly, in 2017, Rajagopal et al. [17] conducted a retrospective analysis on the risk of bleeding after tracheostomy (53% PDT, 47% surgical tracheostomy) in 978 patients. A total of 53% of the patients were on at least one anticoagulation (coumadin, UFH, or low-molecular-weight heparin) or APT (aspirin or clopidogrel). They found out that 4.2% of patients on at least one anticoagulant and/or APT and 3.7% of patients not on any anticoagulant and/or APT experienced bleeding (*p* = 0.698). They concluded that “there is no significant post-operative bleeding risk with the use of any of the common anti-coagulants while undergoing either a percutaneous or surgical tracheostomy. (…) This allows for tracheostomy procedures to be performed immediately and avoidance of delays due to holding/reversing anti-coagulation” [17].

There exist contrary results regarding the impact of anticoagulation and/or APT on the bleeding risk when performing a PDT. Abouzgheib et al. and Nam et al. stated that there is no statistically significant difference in (minor) bleeding rates between patients who were receiving APT or not (*p* = 0.85 and *p* = 0.657) [22,23]. On the other hand, Noy et al. found out that patients treated with DAPT or SAPT compared with those without APT had an increased odds ratio for early minor but not major bleeding (4.22 and 1.87) [21]. Moreover, Huang and et al. concluded that patients who were on APT or anticoagulation regularly before PDT had a higher bleeding risk than patients without (27% vs. 7%, OR: 4.93, *p* < 0.001) [15]. Furthermore, a meta-analysis of 14 studies found out that DAPT (OR: 2.05, *p* = 0.01) but not SAPT or anticoagulation was significantly associated with an increased bleeding risk in PDT patients. However, the “methodological quality was poor” [59].

It is not easy to transform all these results into clinical recommendations. APT and/or anticoagulation appear to be associated with an increased risk of bleeding when performing a PDT, but only with minor bleedings which are usually of little clinical relevance. This would suggest that it generally may not be necessary to pause this medication because the slightly increased risk of bleeding may be acceptable. Nevertheless, the risk of discontinuation (thrombosis or thromboembolism) or continuation (bleeding) should always be weighed up individually. Moreover, it should be noted that none of the above-mentioned studies mentioned a bleeding-associated death in the context of PDT. Overall, PDT appears to be safe to do even in patients with APT and/or anticoagulation. However, further high-quality studies are needed for more well-founded recommendations.

### 4.2. The Influence of Different Single-Step PDT Techniques on the Bleeding Risk

The special feature of this study is the comparison of two single-step PDT techniques (direct and indirect PDT) with regard to differences in the frequency of complications. To the best of our knowledge, there have not been any studies conducted on this matter.

In the present study direct PDTs were performed in 33% of patients and indirect PDTs were performed in 67%. Indirect PDT was performed less frequently in “APT patients” (62% vs. 71%). This distribution was coincidental and was based on the fact that two interventionalists performed the indirect technique and only one interventionalist performed the direct technique.

Periprocedural airway and skin bleeding were more frequent in indirect PDTs (52% and 14%) than direct PDTs in “no APT patients” (32% and 0%, *p* = 0.04 and *p* = 0.02, Table 5). In “APT patients” a significant difference was only seen in periprocedural airway bleedings (69% vs. 45%, *p* = 0.01). However, there were also insignificantly more frequent periprocedural skin bleedings (16% vs. 6%, *p* = 0.11) in “APT patients” undergoing indirect PDTs (Table 5). Moreover, periprocedural airway bleedings were more frequent in “APT patients” when performing an indirect PDT (69% vs. 52%, *p* = 0.02, Table 5). There was no significant difference in the death in ICU or hospital rate, the days from PDT to death, postprocedural bleeding, tracheal cartilage fractures, mean pRBC units required after PDT, the need for bronchoscopic re-intervention, or fever on procedure day and one or three days after PDT (Table 5). To conclude, indirect PDTs appear to generally increase the risk of (clinically irrelevant) periprocedural airway and possibly skin bleedings, especially in APT patients. This in line with our own personal clinical experience. We suspected that the indirect technique was more invasive as associated bleedings seemed to be more difficult to control because of the larger skin incisions and wound areas.

### 4.3. Limitations

Despite the present study being the first one to compare two single-step PDT techniques with regard to differences in complications it also has some limitations, all of which could decrease its external validity and complicate transferability to the general population. Conducting the study in a unicentre retrospective design could have led to potential biases (e.g., selection bias) and the lack of randomization and blinding may have caused confounders. In addition, the limited number of patients included may have led to the study being underpowered to prove further differences in complication frequency between indirect and direct PDT. In addition, all PDTs were performed by experienced interventionalists and so the influence of operator skills could not be considered.

## 5. Conclusions

PDTs appear to be safe in patients receiving APT. Nevertheless, the risk of discontinuation or continuation of APT should always be weighed up individually. Indirect PDTs appear to generally increase the risk of clinically irrelevant, minor periprocedural airway and possibly skin bleedings, especially in APT patients.

## Figures and Tables

**Table 1:** Clinical data.

	“No APT Patients” *n* = 135 (49%)	“APT Patients” *n* = 138 (51%)	All Patients *n* = 273 (100%)	*p*-Value
Sex, female, *n* (%)	57 (42%)	43 (31%)	100 (37%)	0.06 ^1^
Age, years, mean ± SD	66 ± 15	69 ± 10	68 ± 13	0.10 ^2^
SAPT *, *n* (%)	0 (0%)	92 (67%)	92 (34%)	**<0.001 ^3^**
DAPT *, *n* (%)	0 (0%)	46 (34%)	46 (17%)	
Prophylactic anticoagulation *, *n* (%)	88 (65%)	74 (54%)	162 (59%)	0.064 ^1^
Therapeutic anticoagulation *, *n* (%)	2 (4%)	4 (7%)	6 (6%)	0.68 ^1^

Legend: * on the day of PDT procedure, APT: antiplatelet therapy, DAPT: dual APT, SAPT: single APT, SD: standard deviation; tests used: ^1^ Fisher’s exact test for count data test; ^2^ Wilcoxon test; ^3^ Mantel–Haenszel Chi-squared test with continuity correction.

**Table 2:** Procedural data.

	“No APT Patients” *n* = 135 (49%)	“APT Patients” *n* = 138 (51%)	All Patients *n* = 273 (100%)	*p*-Value
PDT, *n* (%)				0.15 ^1^
Direct	41 (29%)	53 (38%)	94 (33%)	
Indirect	94 (71%)	85 (62%)	179 (67%)	
Ventilation duration until PDT, days, median, and IQI	12 (9–16)	12 (9–16)	12 (9–16)	0.77 ^2^
Total ventilation duration, days, median, and IQI	36 (28–53)	34 (28–46)	35 (28–50)	0.32 ^2^

Legend: APT: antiplatelet therapy, PDT: percutaneous dilatational tracheostomy, SD: standard deviation, IQI: interquartile interval; tests used: ^1^ Fisher’s exact test for count data test; ^2^ Wilcoxon test.

**Table 3:** Laboratory data.

	“No APT Patients” *n* = 135 (49%)	“APT Patients” *n* = 138 (51%)	All Patients *n* = 273 (100%)	*p*-Value
Hemoglobin, g/dL, mean ± SD				
On procedure day	9.5 ± 1.5	8.9 ± 1.3	9.2 ± 1.4	**0.004**
One day after	9.3 ± 1.5	9.1 ± 1.3	9.2 ± 1.4	0.71
Three days after	9.3 ± 1.2	8.9 ± 1.0	9.1 ± 1.1	**0.02**
Platelet count, G/L, mean ± SD				
On procedure day	320 ± 182	279 ± 126	299 ± 157	0.13
One day after	326 ± 185	289 ± 120	307 ± 157	0.24
Three days after	337 ± 186	308 ± 122	322 ± 157	0.62
Urea, mg/dL, mean ± SD				
On procedure day	62 ± 41	76 ± 44	69 ± 43	**<0.001**
One day after	57 ± 38	71 ± 46	64 ± 43	**<0.001**
Three days after	54 ± 34	65 ± 37	59 ± 36	**<0.001**
Quick, %, mean ± SD				
On procedure day	79 ± 15	78 ± 14	78 ± 15	0.58
One day after	77 ± 14	75 ± 14	76 ± 14	0.43
Three days after	76 ± 14	74 ± 14	75 ± 14	0.09
INR, mean ± SD				
On procedure day	1.16 ± 0.13	1.16 ± 0.12	1.16 ± 0.13	0.56
One day after	1.17 ± 0.11	1.18 ± 0.14	1.18 ± 0.12	0.48
Three days after	1.17 ± 0.13	1.20 ± 0.13	1.19 ± 0.13	0.09
aPTT, s, mean ± SD				
On procedure day	29.4 ± 9.9	31.3 ± 13.9	30.4 ± 12.1	0.41
One day after	29.2 ± 9.1	31.2 ± 11.5	30.2 ± 10.4	0.38
Three days after	30 ± 11	31 ± 13	30 ± 12	0.78

Legend: APT: antiplatelet therapy, aPTT: activated partial thromboplastin time, INR: international normalized ratio, SD: standard deviation; test used: Wilcoxon test.

**Table 4:** Complications.

	“No APT Patients” *n* = 135 (49%)	“APT Patients” *n* = 138 (51%)	All Patients *n* = 273 (100%)	*p*-Value
Death, *n* (%)				
In ICU,	22 (16%)	29 (21%)	51 (19%)	0.35 ^1^
In hospital	7 (5%)	11 (8%)	18 (7%)	0.47 ^1^
Days from PDT to death, median and IQI	30 (21–65) *n* = 29	30 (13–53) *n* = 40	30 (15–63) *n* = 69	0.50 ^2^
Periprocedural airway bleeding, *n* (%)	61 (46%)	82 (60%)	143 (53%)	**0.03** ^1^
Periprocedural skin bleeding, *n* (%)	13 (10%)	17 (12%)	30 (11%)	0.56 ^1^
Postprocedural bleeding, *n* (%)	2 (1%)	2 (1%)	4 (1%)	1.00 ^1^
Tracheal cartilage fracture, *n* (%)	51 (39%)	53 (39%)	104 (39%)	1.00 ^1^
Need for pRBCs after PDT, *n* (%)	40 (30%)	54 (39%)	94 (34%)	0.13 ^1^
Need for bronchoscopic re-intervention, *n* (%)	0 (0%)	3 (2%)	3 (1%)	0.25 ^1^
Fever on PDT, *n* (%)				
Procedure day	32 (24%)	45 (33%)	77 (28%)	0.11 ^1^
One day after	48 (36%)	60 (43%)	108 (40%)	0.22 ^1^
Three days after	30 (22%)	33 (24%)	63 (23%)	0.77 ^1^

Legend: APT: antiplatelet therapy, ICU: intensive care unit, PDT: percutaneous dilatational tracheostomy, pRBCs: packed red blood cells, SD: standard deviation; IQI: interquartile interval; tests used: ^1^ Fisher’s exact test for count data test; ^2^ Wilcoxon test.

**Table 5:** Comparison of PDT techniques and APT cohorts.

	No APT Patients			APT Patients			No APT vs. APT Patients	
	Direct PDT (*n* = 38, 29%)	Indirect PDT (*n* = 94, 71%)	*p*-Value	Direct PDT (*n* = 51, 38%)	Indirect PDT (*n* = 85, 62%)	*p*-Value	Direct PDT *p*-Value	Indirect PDT *p*-Value
**Death in ICU, *n* (%)**	7 (18%)	15 (16%)	0.80	14 (27%)	15 (18%)	0.2	0.45	0.84
**Death in hospital, *n* (%)**	3 (8%)	4 (4%)	0.41	4 (8%)	6 (7%)	1	1	0.52
**Days from PDT to death, median (IQI)**	29 (24–61)	41 (18–69)	0.84	34 (21–68)	25 (12–48)	0.38	0.91	0.42
**Periprocedural airway bleeding, *n* (%)**	12 (32%)	49 (52%)	**0.04**	23 (45%)	59 (69%)	**0.01**	0.27	**0.02**
**Periprocedural skin bleeding, *n* (%)**	0 (0%)	13 (14%)	**0.02**	3 (6%)	14 (16%)	0.11	0.26	0.68
**Postprocedural bleeding, *n* (%)**	1 (3%)	1 (1%)	0.49	0 (0%)	2 (2%)	0.53	0.43	0.60
**Tracheal cartilage fracture, *n* (%)**	16 (42%)	35 (37%)	0.69	17 (33%)	36 (42%)	0.36	0.51	0.54
**Need for pRBCs**	11 (29%)	28 (30%)	1	20 (39%)	32 (38%)	0.86	0.37	0.27
**pRBC units required after PDT, mean ± SD**	2.55 ± 1.29	2.25 ± 0.75	0.57	2.30 ± 0.98	2.12 ± 0.66	0.50	0.53	0.48
**Need for bronchoscopic re-intervention, *n* (%)**	0 (0%)	0 (0%)	1.00	2 (4%)	1 (1%)	0.86	1	1
**Fever on PDT procedure day, *n* (%)**	10 (26%)	22 (23%)	0.82	18 (35%)	27 (32%)	0.71	0.49	0.24
**Fever one day after PDT, *n* (%)**	12 (32%)	35 (37%)	0.69	25 (49%)	34 (40%)	0.37	0.13	0.76
**Fever three days after PDT, *n* (%)**	5 (13%)	24 (26%)	0.16	13 (26%)	20 (24%)	0.84	0.18	0.86

Legend: APT: antiplatelet therapy, ICU: intensive care unit, IQI: interquartile interval, PDT: percutaneous dilatational tracheostomy, pRBCs: packed red blood cells, SD: standard deviation; test used: for metric values: Wilcoxon test, for categorial: Fisher’s exact test for count data.

## Data Availability

The original contributions presented in the study are included in the article. Further inquiries can be directed to the corresponding author.
